# miR-200a-3p promotes the malignancy of endometrial carcinoma through negative regulation of epithelial-mesenchymal transition

**DOI:** 10.1007/s12672-024-01106-w

**Published:** 2024-06-25

**Authors:** Ying Ma, Yiru Wang, Can Wang, Yan Wang, Jingshu Hu, Zexue Zhang, Tuo Dong, Xiuwei Chen

**Affiliations:** 1 Department of Gynecology Oncology, Harbin Medical University Cancer Hospital, No. 150 Haping Road, Harbin, 150081 Heilongjiang China; 2 Department of Hygienic Microbiology, Public Health College, Harbin Medical University, No. 157 Baojian Road, Harbin, 150081 Heilongjiang China

**Keywords:** miR-200a-3p, Epithelial mesenchymal transition, Endometrial carcinoma, Zinc finger E-box binding homeobox 1

## Abstract

**Background:**

miR-200a-3p is involved in the progression of malignant behavior in various tumors, and its mechanism of action in endometrial cancer is speculated to be related to epithelial-mesenchymal transition (EMT). Therefore, this study explored the metastatic mechanism of miR-200a-3p and EMT in endometrial cancer, with the aim of identifying potential therapeutic targets.

**Methods:**

qRT-PCR was used to analyze miR-200a-3p expression in HEC-1B and Ishikawa cell lines. The cell proliferation assay, transwell assay, and cell scratch test were used to assess changes in the malignant phenotypes of cells after regulating miR-200a-3p expression. Changes in EMT-related protein zinc finger E-box binding homeobox 1 (ZEB1) were detected after regulating miR-200a-3p expression. An endometrial carcinoma transplantation mouse tumor model was constructed, and multiple EMT-related proteins were examined.

**Results:**

The expression of miR-200a-3p and ZEB1 in the endometrial cancer cell lines was higher than in normal endometrial epithelial cell lines (P < 0.05). After silencing miR-200a-3p, the expression of EMT-related protein ZEB1 increased, indicating a negative correlation. Simultaneously, the proliferation, invasion, and metastasis of endometrial cancer cells were significantly enhanced. After miR-200a-3p overexpression, the corresponding malignant phenotype was reversed (P < 0.05). In in vivo experiments, the degree of tumor malignancy and the expression level of EMT-related proteins were significantly reduced in the miR-200a-3p mimic group (P < 0.05).

**Conclusion:**

This study found that miR-200a-3p is a promising target, regulating the EMT process and promoting endometrial cancer progression.

**Supplementary Information:**

The online version contains supplementary material available at 10.1007/s12672-024-01106-w.

## Introduction

In recent years, the overall incidence of endometrial cancer and incidence in younger ages have increased [1]. According to 2020 statistics from the International Agency for Research on Cancer (IARC), the global number of new cases of endometrial cancer reached 417,367. It is second only to cervical cancer incidence, ranking sixth in the incidence of female malignant tumors, and thus seriously threatens the lives and health of women [2]. IARC data revealed that the number of deaths due to endometrial cancer was nearly 100,000, with distant metastasis being the leading cause of death [3]. The five-year survival rate of patients with distant metastasis was reported to be only 17% [4]. Despite continuous improvements in surgical skills in recent years, the mortality rate of endometrial cancer patients with distant metastases has not decreased [5]. In the era of personalized medicine, it is necessary to determine the best treatment plan for each patient's specific situation. The introduction of molecular/genomic mapping will help to tailor the most appropriate treatment, especially in advanced/metastatic setting, where molecular characterization provides important information on the most appropriate treatment [6].Therefore, exploring the distant metastasis mechanism of endometrial cancer is imperative. The results could provide potential targets for clinical adjuvant therapy.

Abnormal characterization of microRNA is closely associated with the occurrence and development of tumors and is involved in various pathophysiological processes [7, 8]. miR-200a-3p is a miR-200a family member and exists within the genetic spectrum of various tumors. It targets the 3’UTR non-coding region to regulate the tumor-promoting or tumor-inhibiting effects of downstream targets [9, 10]. WU [11] demonstrated that miR-200a-3p enhanced the proliferation and metastasis of non-small cell lung cancer cells by targeting SOX17 downregulation. Lv [12]observed that SNHG10 could up-regulate BIN1 by sponging miR-200a-3p through tumor suppression in epithelial ovarian cancer (EOC), thereby inhibiting the occurrence of EOC and epithelial-mesenchymal transition (EMT). By inhibiting the expression of miR-200a-3p, the expression of EMT-related targets, such as N-cadherin, Vimentin and Alpha-SMA, can be significantly enhanced, while the expression of E-Cadherin can be decreased. However, no study has elucidated its mechanism of action in endometrial cancer.

Our research group explored the role of miR-200a-3p in endometrial cancer to identify possible downstream regulatory targets and clarify the mechanism causing distant metastasis in endometrial cancer. Recent studies reported that EMT, a complex biological process, could promote cancer progression and distant metastasis [13, 14]. EMT process inhibitors can significantly inhibit the invasion and metastasis of breast, bladder, and other tumors [15, 16]. Therefore, we explored the correlation between miR-200a-3p and EMT. Zinc finger E-box binding homeobox 1 (ZEB1) is closely related to EMT and has noticeable phenotype changes after regulating miR-200a-3p [17].

ZEB1 is a zinc finger homologous transcription factor family member located on chromosome 10 (10p11.2). It can promote tumor cell activity through EMT and enhance the migration and invasion of tumor cells. ZEB1 can reconstruct the tumor microenvironment and plays a synergistic role as a multi-effect transcription factor [18]. Colangelo [19]reported that ZEB1 enhanced the aggressiveness of colorectal cancer cells by promoting the EMT process and driving the pathological process behind the colorectal cancer malignant phenotype. Wu HT also revealed that ZEB1 had mesenchymal properties, increasing the proliferation, metastasis, and drug resistance of breast cancer cells [20]. Diepenbruck [21]incorporated microRNA sequencing and functional verification in the mouse model of metastatic breast cancer to determine the function of miR-1199-5p and ZEB1 in the double-negative feedback loop, thereby controlling the occurrence of EMT and tumor metastasis. ZEB1 is similar to E-Cadherin in the EMT process and is the target gene of miR-200a-3p. miR-200a-3p can directly target part of ZEB1, which has the potential to be used in the therapy of endometrial cancer.

**Fig. 1:**
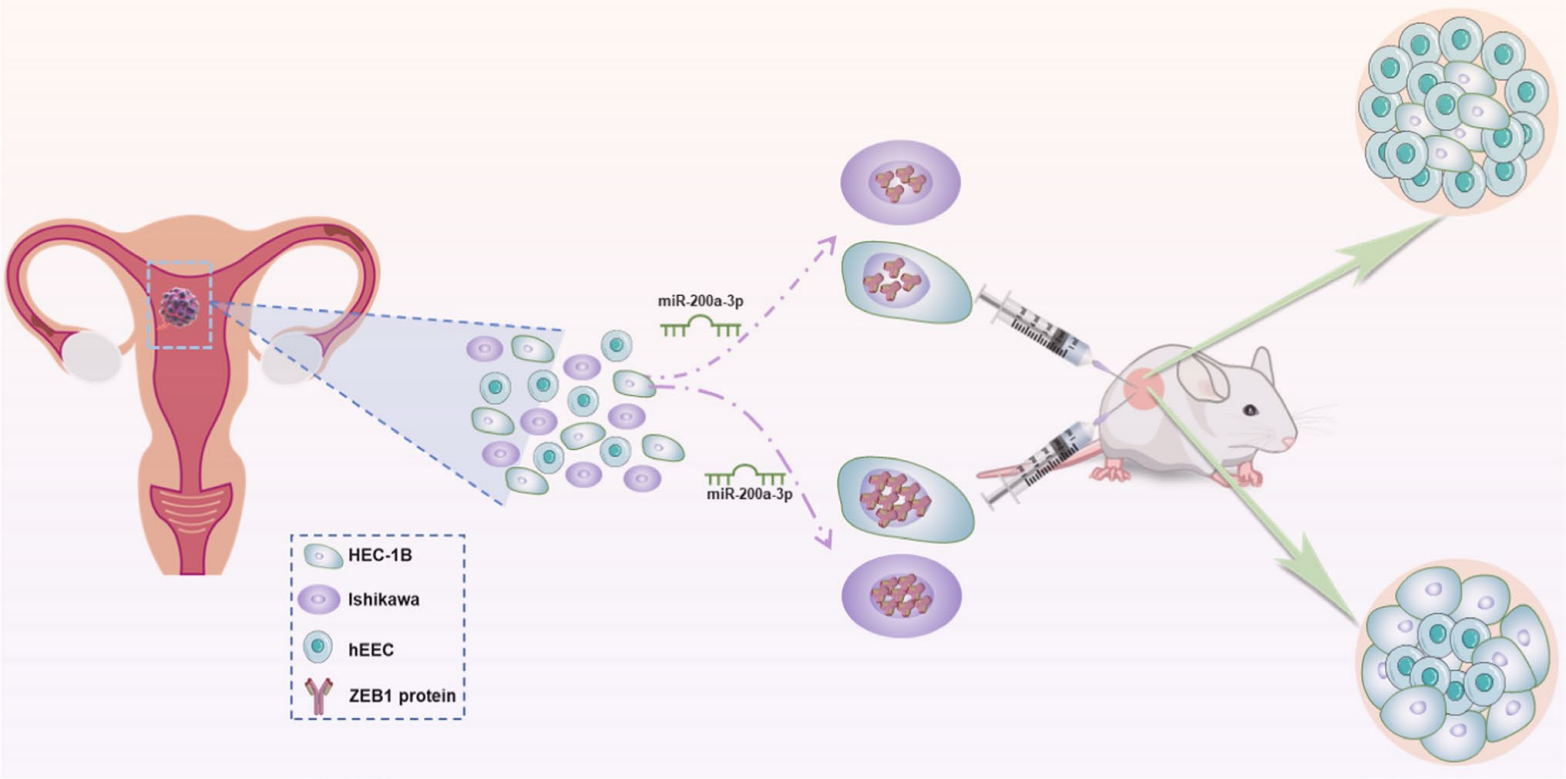
The schematic diagram of this study

Therefore, the current study selected miR-200a-3p to investigate the role of abnormal microRNA expression in endometrial cancer and the resulting tumor malignancy. The study attempted to clarify the mechanism of miR-200a-3p in endometrial cancer invasion and metastasis and other EMT-related malignant phenotypes through in vitro and in vivo experiments (Fig. 1) to provide more novel targeted treatment options for patients with distant metastases of advanced endometrial cancer.

## Materials and methods

### Cell lines and cell culture

The Department of Epidemiology, School of Public Health, Harbin Medical University donated the endometrial cancer cell lines (HEC-1B and Ishikawa) and the normal endometrial hEEC cell line. HEC-1B cells were cultured in Minimal Essential Medium (MEM), and Ishikawa and hEECs were cultured in Dulbecco’s modified Eagle medium (DMEM). Fetal bovine serum (10%) and 1% cyan-streptomycin were added to each medium. Insulin (10 μg/mL) and endothelial growth factor (5 ng/mL) were added to DMEM used for culturing hEECs. All cells were cultured in a cell incubator at 37 ℃ and 5% CO_2_.

### Quantitative real-time PCR

Total RNA was extracted using an animal RNA extraction kit (Beyotime, R0026). PrimeScript™ RT Master Mix kit (Takara, RR036A) and 2 × SYBR Green qPCR Master Mix (Bimake, B21203) were used to perform quantitative fluorescence PCR. The primer sequences of miR-200a-3p and ZEB1 are shown in Supplementary Table 1. The reaction conditions were 95 ℃ for 15 s, 60 ℃ for 30 s, and 72 ℃ for 30 s for 40 cycles.

### Western blots

Nuclear proteins were extracted with a nucleoprotein extraction kit (Solarbio, R0050). The BCA method was used analyze protein concentrations by comparison with a standard curve. Sodium dodecyl sulfate-gel electrophoresis was performed after the appropriate loading amount was determined. Then, the membrane was transferred, closed, and incubated with ZEB1 monoclonal antibody (ABclonal, A21794) at 4 ℃ overnight. Finally, the secondary antibody was added and incubated for 1 h. The protein bands were observed using ECL reagent.

### Cell transfection with miR-200a-3p-overexpressing and knocked down microRNA mimic

The miR-200a-3p-overexpressing mimic (miR-200a-3p mimic), miR-200a-3p knocked down mimic (miR-200a-3p inhibitor), and control blank (NC) were developed by GenePharma, China. About 80% of the fused cells were transfected, and Lipo3000 was diluted using opti-MEM. Transfection efficiency was measured after 72 h of transfection.

### Cell proliferation, invasion, and metastasis experiments

The proliferation of endometrial cancer cells transfected with miR-200a-3p inhibitor and mimic were measured using a CCK8 kit (Beyotime, C0037). After cell lamination using 96-well plates, miR-200a-3p knockdown and overexpressed mimic were transfected with Lipo3000 reagent for further culture. Then, 10 μL of CCK8 solution was added to each well, and absorbance was measured at 450 nm. Cells in the logarithmic growth stage were cultured without serum starvation and inoculated into a transwell chamber at a density of 4 × 10^5^ cells. After staining and fixation, cell invasion was observed under a microscope. In the cell scratch experiment, six-well plates helped in cell planking, and 5 × 10^5^ endometrial cancer cells were added to each hole for the 24 h culture.

### Animal experiment

Fifteen five-week-old BALB/C female nude mice weighing 20–25 g were purchased from Charles River, China, and grouped into miR-200a-3p inhibitor, mimic, and blank groups (n = 5). HEC-1B endometrial cancer cells were transfected with the miR-200a-3p inhibitor, mimic, and blank control, and 2 × 10^6^ cells were implanted subcutaneously in each mouse. All mice were provided free access to water and food and maintained at 20 ℃ and 50% constant humidity. The mice were observed daily. The mice were euthanized after two weeks of tumor formation, and the tumors were extracted and weighed for further experiments.

### Immunohistochemistry

Mice tumor grafted tissues were embedded in paraffin, sectioned, and dewaxed in xylene, hydrated using different ethanol concentrations, and repaired with the H_2_O_2_ antigen. Finally, the tissues were incubated with ZEB1 primary and secondary antibodies (ABclonal, A5600) and stained with DAB and hematoxylin. and the tablets were sealed after dehydration for observation. Then, the degree of staining was quantified and photographed using a microscope.

### Enzyme-linked immunosorbent assay

The fibronectin (FN) and Snail protein enzyme-linked immunosorbent assay (ELISA) kits were purchased from BYabscience, China. The enzyme-labeled reagent, chromogenic agent, and the reaction terminator in the kit were used for corresponding incubation at 37℃. The absorption value was determined at 450 nm after reaction termination.

### Statistical analysis

SPSS 19 was used for all statistical analyses. The data are expressed as the mean ± standard deviation. The t-test was used to analyze statistical differences between the control and experimental groups, and P-values of < 0.05 were considered statistically significant. Each experiment was repeated three times.

## Results

### miR-200a-3p and ZEB1 are highly expressed in endometrial cancer cell lines

**Fig. 2:**
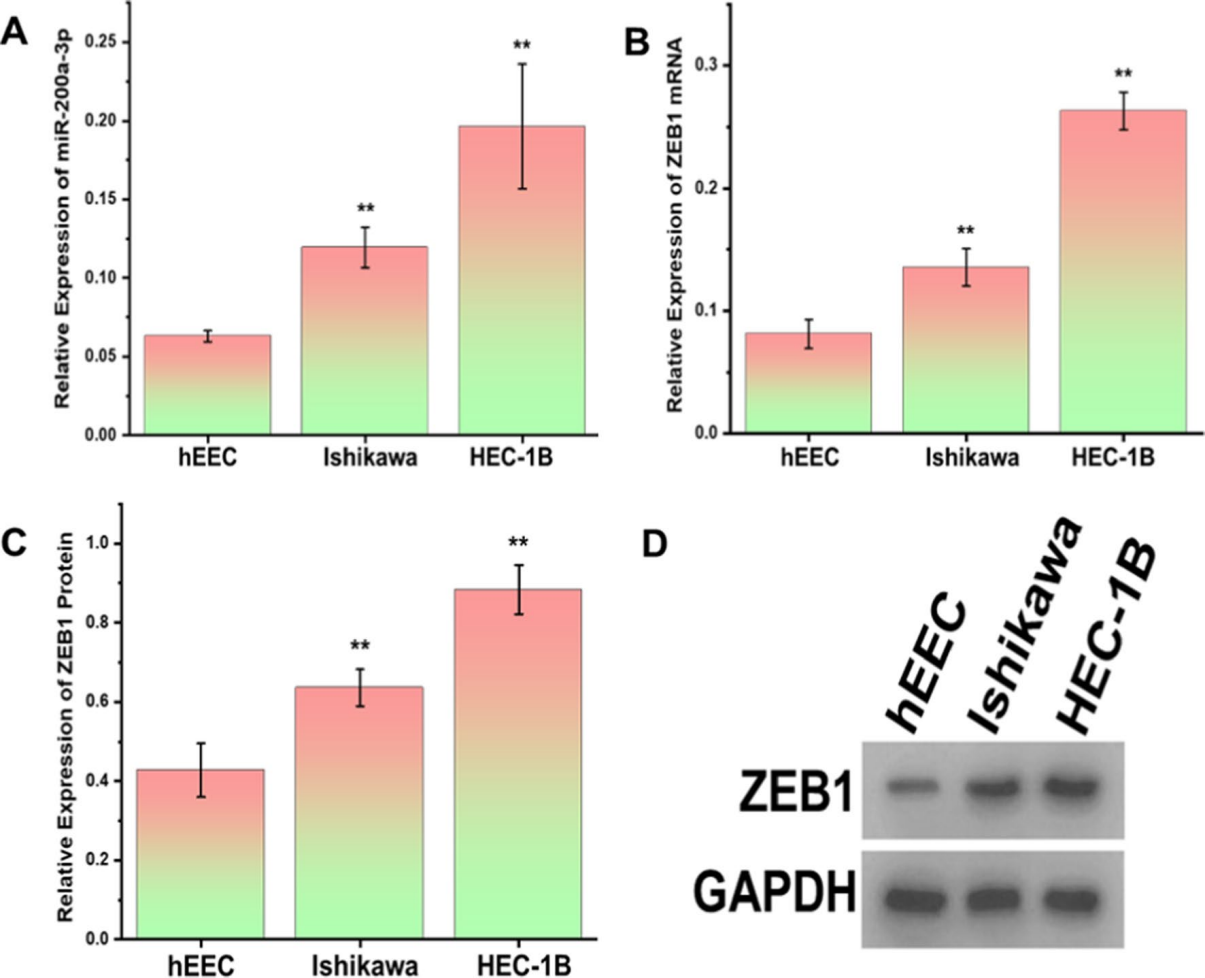
miR-200a-3p and ZEB1 were highly expressed within the endometrial cancer cell lines. **A** The qRT-PCR detection of miR-200-3p in endometrial cancer cell lines and normal endometrial cell lines. **B** The relative ZEB1 mRNA expression in endometrial cancer cell lines and normal endometrial cell lines. **C**, **D** The relative ZEB1 protein expression in Ishikawa and HEC-1B cells and hEEC cells. **P<0.05. All the experiments were repeated thrice

The qRT-PCR results revealed that miR-200a-3p expression was significantly up-regulated in the HEC-1B and Ishikawa endometrial epithelial cell lines compared to the normal hEEC endometrial epithelial cell line (Fig. 2A; **P < 0.05). This was consistent with the expression of bioinformatics database analysis in endometrial cancer patients (Fig. S1). ZEB1 mRNA expression was higher in the endometrial cancer cell lines than in the normal endometrial epithelial cell line (Fig. 2B; **P < 0.05). The Western blot results revealed that ZEB1 protein expression levels were significantly higher in the endometrial cancer cell lines than in normal endometrial epithelial cells (Fig. 2C, D; **P < 0.05).

### MiR-200a-3p is negatively associated with ZEB1 expression in endometrial cancer

**Fig. 3:**
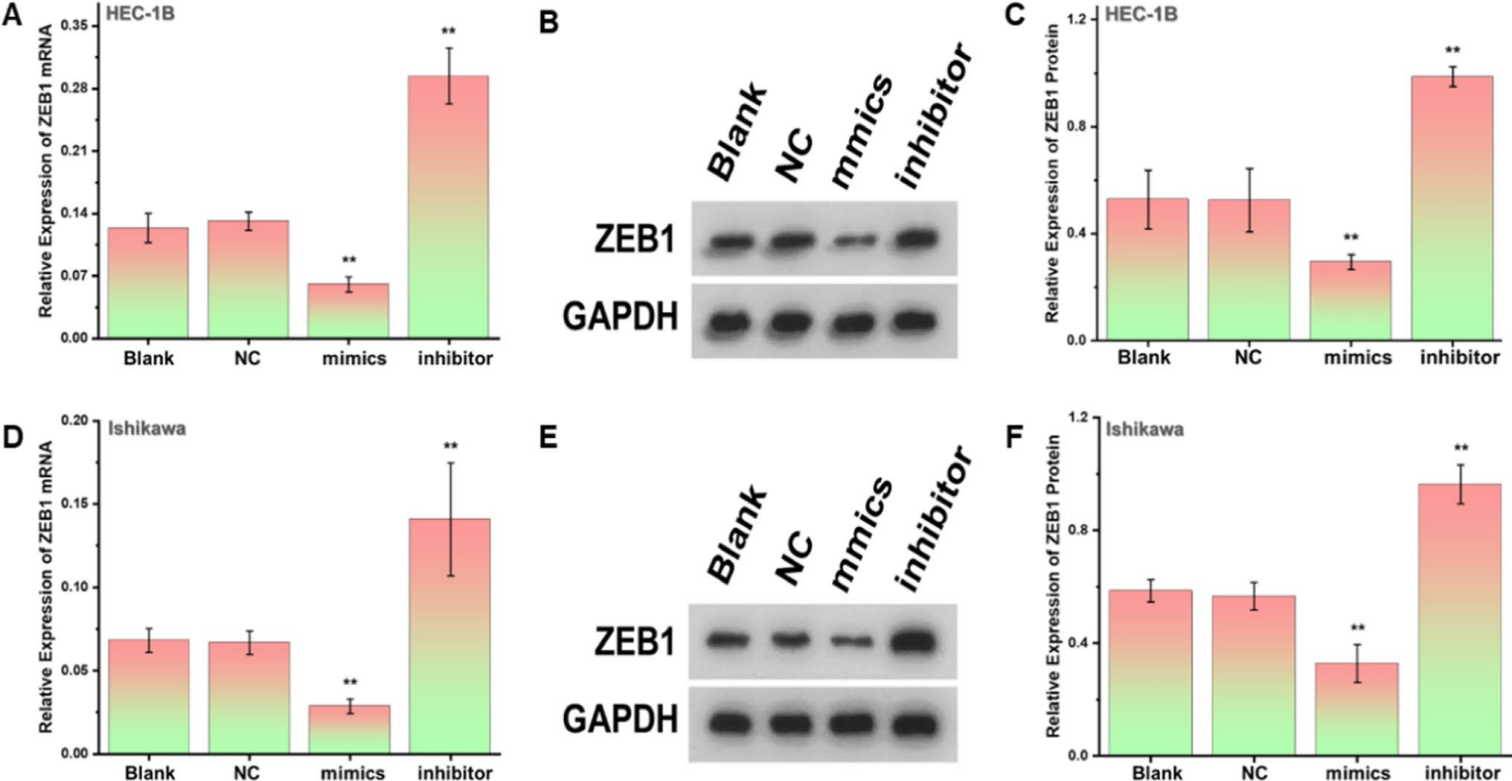
miR-200a-3p was negatively correlated with ZEB1 expression in endometrial cancer. **A** After MIR-200a-3p knockdown, the ZEB1 mRNA expression level was significantly elevated by the qRT-PCR method in HEC-1B endometrial cancer cell lines.** B**, **C **After the MIR-200a-3p regulation, ZEB1 protein depicted opposite expression.** D–F** The negative correlation between ZEB1 mRNA and protein levels was verified in Ishikawa cell lines after miR-200a-3p regulation. **P<0.05. All the experiments were repeated thrice

qRT-PCR was used to verify the successful knockdown and overexpression of miR-200a-3p in HEC-1B and Ishikawa endometrial cancer cell lines (**Fig. S2A-B**; **P < 0.05). qRT-PCR was also used to analyze changes in ZEB1 in the HEC-1B endometrial cancer cell line at the mRNA expression level after knocking down and overexpressing miR-200a-3p. The results indicated that the expression level of ZEB1 in the HEC-1B endometrial cancer cell line was significantly elevated after the knockdown and overexpression of miR-200a-3p (Fig. 3A; **P < 0.05). The ZEB1 content change at the protein level was consistent with the mRNA level (Fig. 3B, C; **P < 0.05) after verifying the miR-200a-3p regulation with western blot, with similar results in the Ishikawa cell line (Fig. 3D–F; **P < 0.05). The same correlations were seen between the two cell lines using bioinformatics analysis (Fig. S2C). Therefore, miR-200a-3p was negatively correlated with ZEB1 expression in endometrial cancer patients.

### Knockdown of miR-200a-3p promotes the proliferation, invasion, and migration of endometrial *cancer* cells

**Fig. 4:**
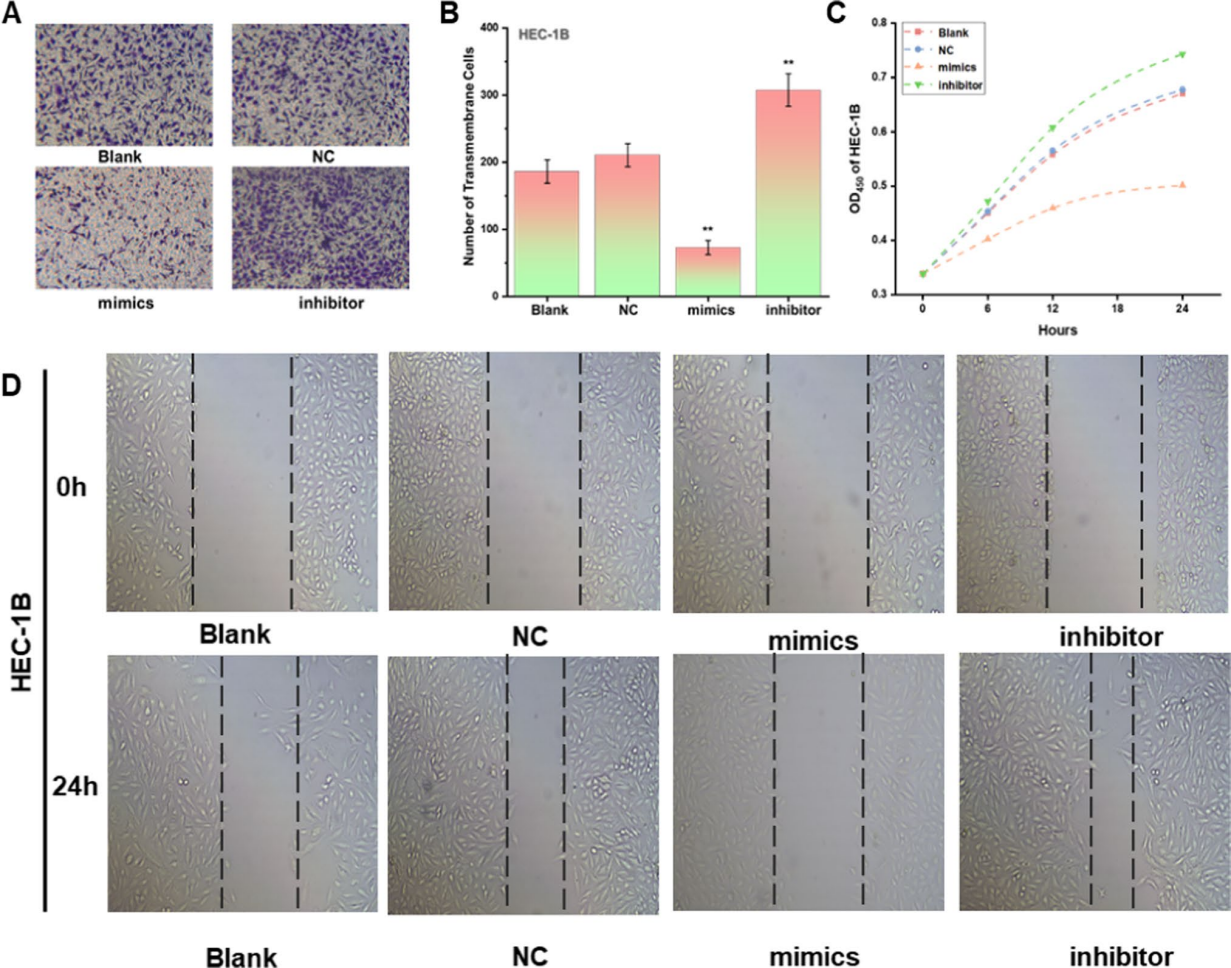
The miR-200a-3p knockdown promoted the proliferation, invasion, and migration of endometrial cancer cells. **A**, **B** The invasive ability of endometrial cancer cells HEC-1B was significantly elevated in miR-200a-3p knockdown group by the Transwell assay.** C **CCK8 assay established that the proliferation of HEC-1B endometrial cancer cells was enhanced after miR-200a-3p knockdown. The OD_450_ value was assessed at 0, 6, 12 and 48 h.** D** Cell migration was compared at 0 h and 24 h using the cell scratch assay, and the migration ability of Mir-knockdown group was significantly elevated in HEC-1B endometrial cancer cells. **P<0.05. All the experiments were repeated thrice

The transwell assay results revealed that the invasion ability of HEC-1B endometrial cancer cells was significantly enhanced in the miR-200a-3p knockdown group. In contrast, the overexpressing group had a lower invasion ability than the control group (Fig. 4A, B). The CCK8 method helped verify the miR-200a-3p knockdown and overexpression effects on the proliferative ability of endometrial cancer cells. The results indicated that the proliferative ability of endometrial cancer cells was increased after miR-200a-3p knockdown and significantly less after overexpression (Fig. 4C). Finally, the cell scratch assay was used to observe changes in cell migration ability after miR-200a-3p knockout. The results depicted the enhanced migration ability of HC-1B endometrial cancer cells within the miR-200a-3p knockout group (Fig. 4D. The same changes in the invasion phenotype were obtained by repeating the test in the Ishikawa endometrial cancer cell line (Fig. S3).

### Overexpression of miR-200a-3p reduces tumorigenicity in mice

**Fig. 5:**
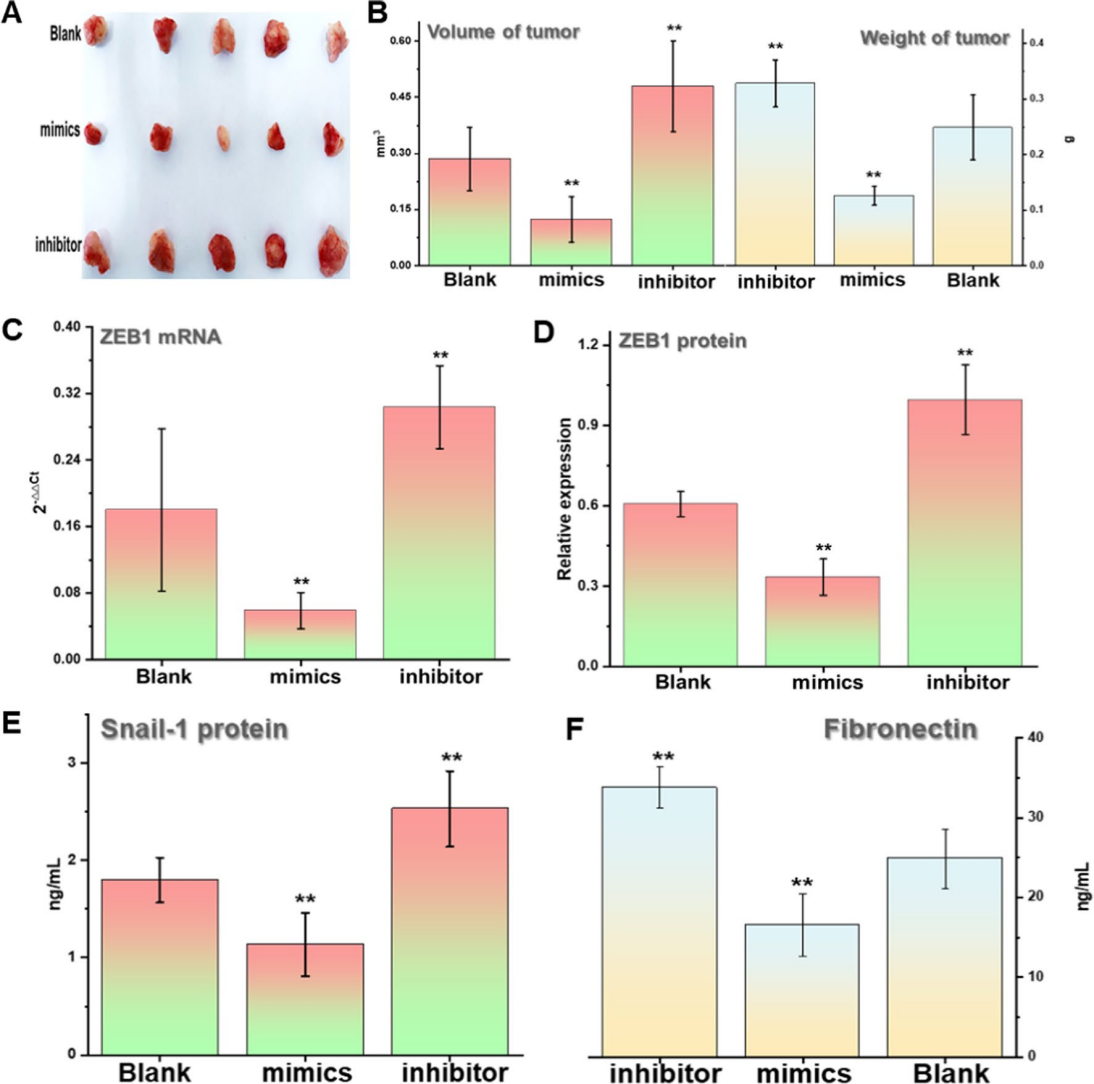
Overexpression of miR-200a-3p reduced the tumorigenicity in mice. **A**, **B** The tumor size and weight of mice overexpressed with miR-200a-3p were significantly smaller than the control group. **C **The mRNA ZEB1 expression level was significantly reduced in mice with miR-200a-3p overexpression and detected using qRT-PCR. **D** Western blot analysis revealed that the ZEB1 protein expression level in the overexpressed miR-200a-3p group was significantly reduced. **E**, **F **Snail protein and fibronectin were decreased in miR-200a-3p overexpressed mice tumor and detected with ELISA. **P<0.05. All the experiments were repeated thrice

We successfully constructed a transplanted mouse tumor model using the HEC-1B endometrial cancer cell line based on the above experimental results. The results indicated that the tumor size and weight of mice transfected with the miR-200a-3p-overexpressing mimic were significantly smaller than the control group (Fig. 5A, B). qRT-PCR and Western blots were used to analyze ZEB1 expression at the mRNA and protein levels in mouse subcutaneous tumors. The results were consistent with our in vitro experimental results in endometrial cancer cell lines.

After regulating miR-200a-3p, ZEB1 showed the opposite expression (Fig. 5C, D). Immunohistochemistry was used to detect changes in ZEB1 expression in tumor tissues with miR-200a-3p overexpression and knockdown. The results also suggested that ZEB1 was primarily located in the nucleus (Fig. S4). Finally, ELISA was used to determine the changes in fibronectin (FN) and SNAIL proteins after regulation to clarify the other EMT-related protein changes in mice tumors after miR-200a-3p overexpression. FN and SNAlL are recognized as EMT-related proteins, depicting the enhanced invasion and metastasis ability of tumor cells [22, 23]. The experimental results indicated that the expression of FN and SNAIL in tumors in the miR-200a-3p-overexpressing group was decreased. In contrast, the expression in the miR-200a-3p-knockout group was high (Fig. 5E-F). Therefore, in vivo experiments in mice demonstrated that miR-200a-3p-overexpression decreased tumorigenicity and attenuated the malignant phenotype of endometrial cancer.

## Discussion

Endometrial cancer has a gradually rising trend of disease load [24]. Despite the combined treatment regimen of standardized surgery with chemotherapy, metastasis and recurrence still occur in many patients. Therefore, exploring the metastatic mechanism of advanced metastatic endometrial cancer and developing more targeted drugs against efficient targets can improve the survival of endometrial cancer patients.

Recently, some scholars conducted relevant studies on the role of miR-200a-3p in non-small cell lung cancer [25], cervical cancer [26], bladder cancer [27], and other tumors, establishing the regulatory mechanism of miR-200a-3p in these tumors. The results showed different mechanisms of action and phenotypic effects of miR-200a-3p in different tumors. We conducted a preliminary exploration of the regulatory role of miR-200a-3p in endometrial cancer and found a significantly high expression level of miR-200a-3p in endometrial cancer cell lines compared to normal endometrial epithelial cells. Simultaneously, knockdown and overexpression regulation of miR-200a-3p depicted a significant decrease in the proliferation, invasion, and migration of endometrial cancer cells after miR-200a-3p up-regulation. In contrast, the corresponding malignant phenotype was significantly enhanced after the down-regulation of miR-200a-3p. Therefore, this preliminary study suggests that miR-200a-3p regulation can negatively regulate endometrial cancer metastasis. Relevant studies reported that the metastasis mechanism of endometrial cancer is closely associated with EMT [28]. EMT is significantly correlated with tumor cell dryness, metastasis potential, immune escape, and treatment resistance [29, 30]. ZEB1 is an efficient transcription factor in the EMT process [31] and is essential for activating the invasion and metastasis phenotypes of endometrial cancer. Therefore, our study explored the correlation between miR-200a-3p and ZEB1. ZEB1 was highly expressed in the endometrial cancer cell lines, consistent with the results of previous studies. Additionally, ZEB1 expression was opposite to miR-200a-3p after its regulation, depicting a negative correlation between them. The mice experiments proved that EMT-related proteins like ZEB1 were significantly elevated after miR-200a-3p knockout. Therefore, miR-200a-3p could regulate MET and indirectly act on the corresponding ZEB1 targets to promote the invasion and metastasis of endometrial cancer. A corresponding mechanistic axis could exist between miR-200a-3p and ZEB1 or competitively bind with other targets [32, 33] to regulate the progression of malignancy. Our research group will explore the specific mechanism producing this effect further.

The corresponding inhibitors were combined with various current efficient delivery systems after elucidating the mechanism of miR-200a-3p in promoting the progression of endometrial cancer. Thus, new delivery materials were developed that could effectively reduce endometrial cancer malignancy and the possibility of distant metastasis while prolonging the survival of patients. Zhou [34] transferred miR-21-5p inhibitors into a mouse lung cancer model using a novel gene delivery technology and conducted ultrasound-targeted microbubble destruction to significantly inhibit lung cancer progression. Ou [35] utilized a graphene oxide-polyethylenimine complex to efficiently deliver miR-214 inhibitors and inhibit the invasion and metastasis of oral squamous cell carcinoma. Liang [36] targeted engineered exosomes to colon cancer cells to deliver a combination of miR-21 inhibitors and 5-fluorouracil, which reversed drug resistance in colon cancer. Liu [37] prepared nanobubbles for loading programmed death ligand 1 (PD-L1) antibody and the miR-424 gene, which significantly enhanced the immunotherapy effect of hepatocellular carcinoma and improved anti-tumor effects. Therefore, the EMT-promoting mechanism of miR-200a-3p and ZEB1 can be used to inhibit the metastatic potential of endometrial cancer, with corresponding effects in immunotherapy and chemotherapy resistance.

This study preliminarily explored the role of miR-200a-3p in endometrial cancer and demonstrated its regulatory role in the EMT process, including ZEB1 expression, which can provide a promising target for treating metastatic endometrial cancer.

### Supplementary Information

Additional file1 (DOCX 6291 KB)

## Data Availability

All relevant data are within the manuscript and its additional files.

## Abbreviations

EMT: Epithelial–mesenchymal transition
ZEB1: Zinc finger E-box binding homeobox 1
IARC: International Agency for Research on Cancer
UTR: Untranslated region
EOC: Epithelial ovarian cancer
FN: Fibronectin
UTMD: Ultrasound-targeted microbubble destruction
PD-L1: Programmed death ligand 1
