# Supplementary material for: miR-200a-3p promotes the malignancy of endometrial carcinoma through negative regulation of epithelial-mesenchymal transition
Source: Discov Oncol. 2024 Jun 25;15:243. doi: 10.1007/s12672-024-01106-w (PMC11199454; doi:10.1007/s12672-024-01106-w)

***Supplementary Information***

**miR-200a-3p promotes the malignancy of endometrial carcinoma through negative regulation of epithelial-mesenchymal transition**

Ying Ma^[a]^, Yiru Wang^[a]^, Can Wang^[a]^, Yan Wang^[a]^, Jingshu Hu^[a]^, Zexue Zhang^[a]^, Tuo Dong^[b]*^, Xiuwei Chen^[a]*^

^[a]^ Department of Gynecology Oncology, Harbin Medical University Cancer Hospital; Haping Road No. 150, Harbin, Heilongjiang Province, 150081, China.

^[b]^ Department of Hygienic Microbiology, Public Health College, Harbin Medical University; Baojian Road No. 157, Harbin, Heilongjiang Province, 150081, China.

*Corresponding Author. E-mail: [chenxiuwei1023@163.com](mailto:chenxiuwei1023@163.com)

[dongtuo@hrbmu.edu.cn](mailto:dongtuo@hrbmu.edu.cn)

[S1. miR-200a-3p expression level with 538 cancer and 33 normal in ENROCI datebase 3](#_Toc165962226)

[S2. miR-200a-3p was successfully knocked down and overexpressed in endometrial cancer cell lines Ishikawa 4](#_Toc165962227)

[S3. Knockdown of miR-200a-3p promoted the proliferation, invasion and migration of endometrial cancer cells Ishikawa 5](#_Toc165962228)

[S4. Overexpression of miR-200a-3p reduced tumorigenicity in mice 6](#_Toc165962229)

[S5. The primer sequence used in this study 7](#_Toc165962230)

[S6. The original blot images 8](#_Toc165962231)

S1. miR-200a-3p expression level with 538 cancer and 33 normal in ENROCI datebase


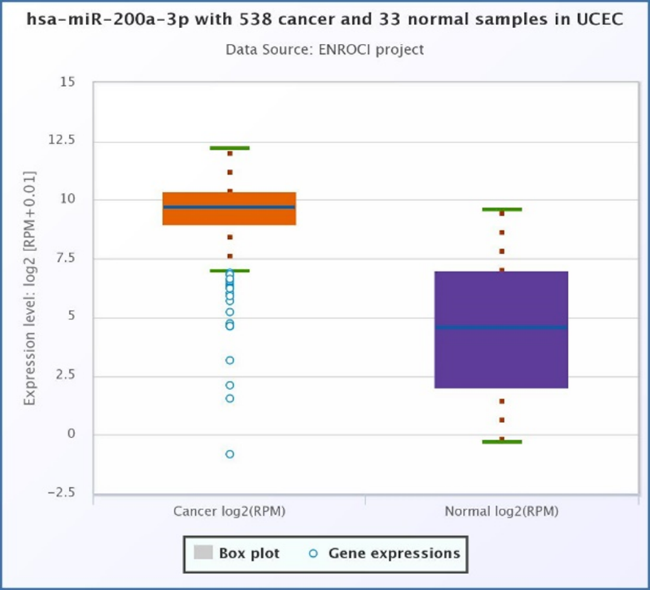


**Fig.S1** miR-200a-3p expression level with 538 cancer and 33 normal in ENROCI datebase.

S2. miR-200a-3p was successfully knocked down and overexpressed in endometrial cancer cell lines Ishikawa


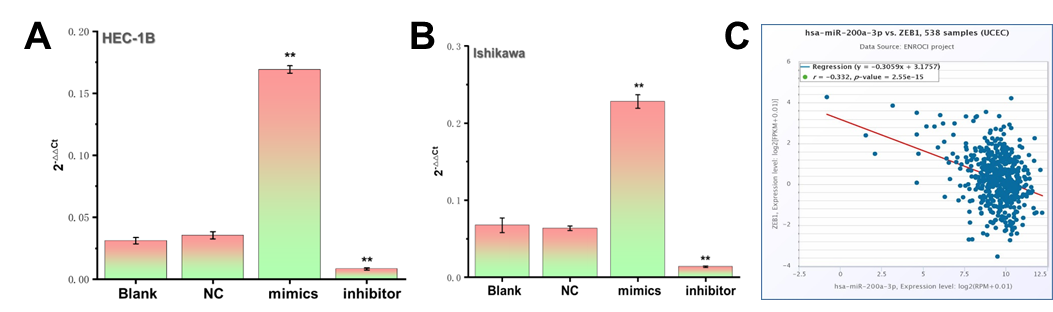


**Fig.S2** miR-200a-3p was successfully knocked down and overexpressed in endometrial cancer cell lines Ishikawa **(A)** and Ishikawa **(B)** by qRT-PCR detection. **(C)** MIR-200a-3p and ZEB1 were negatively correlated with UCEC samples in ENROCI datebase. **P<0.05. All experiments were repeated three times.

S3. Knockdown of miR-200a-3p promoted the proliferation, invasion and migration of endometrial cancer cells Ishikawa


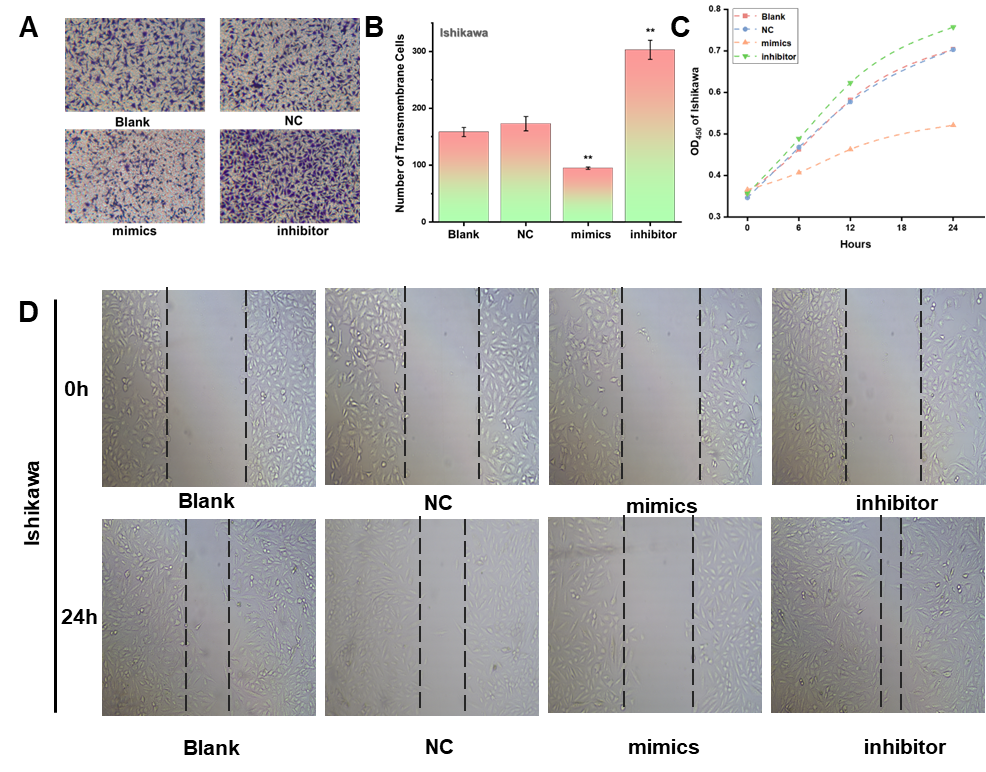


**Fig. S3** Knockdown of miR-200a-3p promoted the proliferation, invasion and migration of endometrial cancer cells. **(A-B)** The invasion ability of endometrial cancer cells Ishikawa was significantly enhanced in miR-200a-3p knockdown group by transwell method. **(C)** CCK8 assay confirmed that the proliferation of Ishikawa endometrial cancer cells was enhanced after knockdown of miR-200a-3p. The value of OD450 was assessed 0, 6, 12 and 48 h. **(D)** Cell migration at 0 h and 24 h was compared by cell scratch assay, and the migration ability of Mir-knockdown group was significantly enhanced in Ishikawa endometrial cancer cells. **P<0.05. All experiments were repeated three times.

S4. Overexpression of miR-200a-3p reduced tumorigenicity in mice


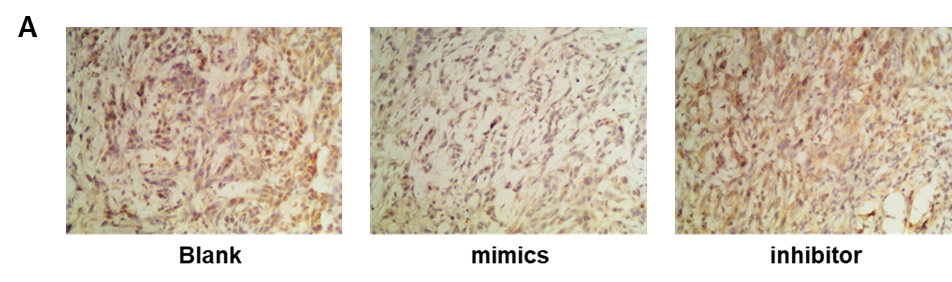


**Fig.S4** Overexpression of miR-200a-3p reduced tumorigenicity in mice. **(A)** Immunohistochemistry showed that ZEB1 was located in the nucleus, and the expression of ZEB1 protein decreased after overexpression of miR-200a-3p.

S5. The primer sequence used in this study

**Table 1. The primer sequence used in this study**

| **Primer Name** | **Primer Sequence (5’to 3’)** |
| --- | --- |
| miR-200a-3p-F | ACAGCACCTCCAACATGCACA |
| miR-200a-3p-R | TGAGTGACAATCCACTTCTACTCCA |
| U6-F | CTCGCTTCGGCAGCACA |
| U6-R | AACGCTTCACGAATTTGCGT |
| ZEB1-F | TCATCGCTACTCCTACTG |
| ZEB1-R | CCTCTTCCCTTGTCAAAC |
| GAPDH-F | CACCCACTCCTCCACCTTTG |
| GAPDH-R | CCACCACCCTGTTGCTGTAG |

S6. The original blot images

**Fig.S5 (A):** The original blot image of Fig.2D; **(B):** The original blot image of Fig.3B; **(C):** The original blot image of Fig.3E.


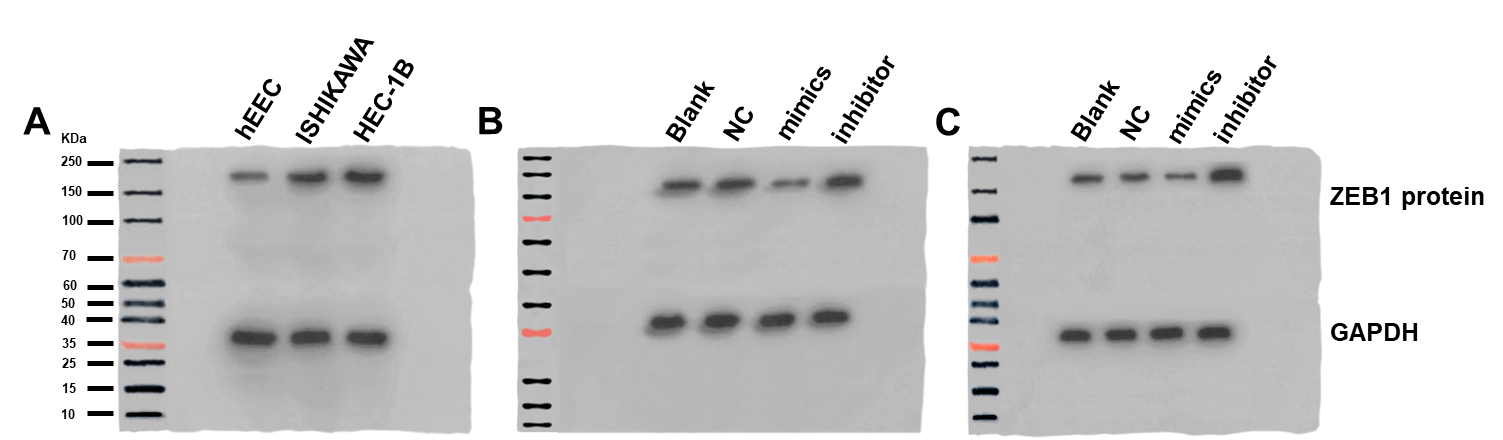

Supplement: Supplementary file 1 — Additional file1 (DOCX 6291 KB) [file 12672_2024_1106_MOESM1_ESM.docx]
